# A sterol-defined system for quantitative studies of sterol metabolism in *C. elegans*

**DOI:** 10.1016/j.xpro.2021.100710

**Published:** 2021-08-10

**Authors:** Benjamin Trabelcy, Yoram Gerchman, Amir Sapir

**Affiliations:** 1 Faculty of Natural Sciences, University of Haifa, Haifa 3498838 Israel

**Keywords:** Genetics, Mass Spectrometry, Metabolism, Model Organisms

## Abstract

This protocol describes the culturing of the nematode *Caenorhabditis elegans (C. elegans)* in a sterol-defined experimental system and the subsequent quantitative analysis of *C. elegans* sterols through gas chromatography-mass spectrometry. Although studied primarily in mammals, sterols are essential biomolecules for most eukaryotes. *C. elegans* cannot synthesize sterols and thus relies on the uptake of dietary sterols. Therefore, *C. elegans* is a powerful system to study metabolism in sterol-defined conditions that are described in our protocol.

For complete details on the use and execution of this protocol, please refer to Shamsuzzama et al. (2020).

## Highlights

• Principles for setting up sterol-defined experimental systems in *C. elegans*
• A protocol for medium-throughput analysis of sterol metabolism by GC-MS
• Strategies for quantitatively determining the levels of sterols in *C. elegans*

## Graphical abstract

**Figure:**
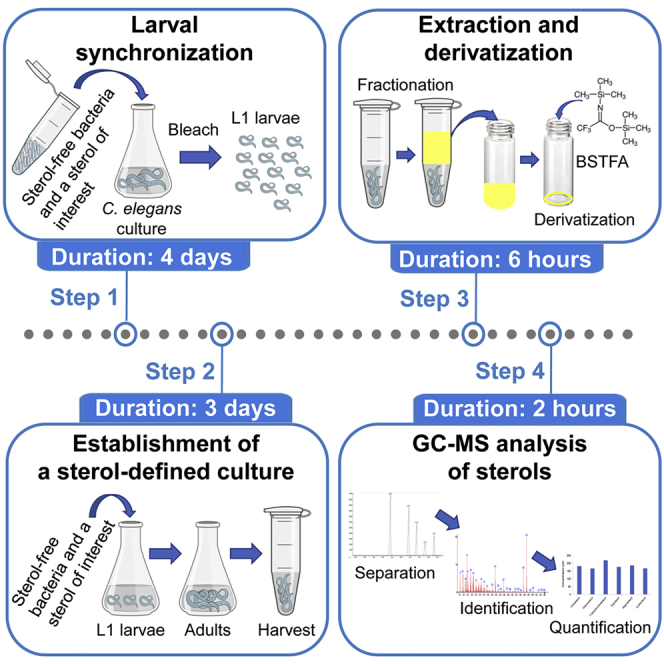

## Before you begin

The unique biology of *C. elegans* makes this nematode a powerful experimental system for the study of sterol metabolism (Shamsuzzama et al., 2020, Capell-Hattam and Brown, 2020, Zhou et al., 2020). *C. elegans* cannot synthesize sterols and thus relies on the uptake of dietary sterols, which are precursors to the synthesis of hormones that coordinate critical stages of *C. elegans* development. Moreover, *C. elegans* can be cultured in a sterol-defined environment in which the only source of sterols is dietary sterols of interest supplemented by the researcher.

a. The principles of establishing sterol-defined cultures.

Before beginning the experiment, the researcher should acquire the mindset of working in a sterol-controlled environment. A typical laboratory setting is usually contaminated with many types of sterols that can affect the experiment—especially because *C. elegans* is a sterol auxotroph that can grow on a very small amount of dietary sterols (Chitwood, 1999, Merris et al., 2003). The researcher should avoid the contamination of solutions and lab equipment (labware) by sterols, including traces of sterols from previous experiments. Therefore, all labware to be used should be new and sterile, and gloves should be used throughout the experiment, including when handling *C. elegans* and its feed bacteria. Any solution or material that is not chemically defined (e.g., yeast extract or Luria Broth medium) should be avoided. Moreover, because most eukaryotes have a significant amount of sterols in their cells (Desmond and Gribaldo, 2009) contamination of the cultures by eukaryotic organisms should be avoided.

b. Adaptation of standard *C. elegans* protocols for sterol-defined studies.

*C. elegans* has its minimal dietary requirements met by a defined growth medium supplemented by sterols (Merris et al., 2004) and bacterial food. Most bacteria, *Escherichia coli* (*E. coli*) included, do not synthesize nor require sterols for living (Wei et al., 2016); thus, the bacterial food of *C. elegans* can be sterol-free. A sterol-defined *C. elegans* culture, however, requires several adaptations of the standard *C. elegans* culture protocol (Stiernagle, 2006). These include: **i)** Working in a sterol-free environment, as described above; **ii)** Growing *C. elegans* in liquid culture rather than on agar plates to avoid the potential contamination of the culture by algal sterols from the ager—a product of red algae. An additional advantage of liquid cultures is the large number of developmentally synchronized animals that can be harvested from such cultures (Heshof et al., 2019), allowing for the analysis of metabolites that are found in small quantities, like sterols (Merris et al., 2004, Yilmaz et al., 2020); **iii)** Growing feed bacteria in a defined (minimal) media, such as Minimum Essential Medium (MEM). MEM does not contain any sterol, in contrast to the commonly used Luria Broth (LB) medium, which includes yeast extract and thus is a potential source of contamination by the yeast sterol ergosterol.

**Figure 1:**
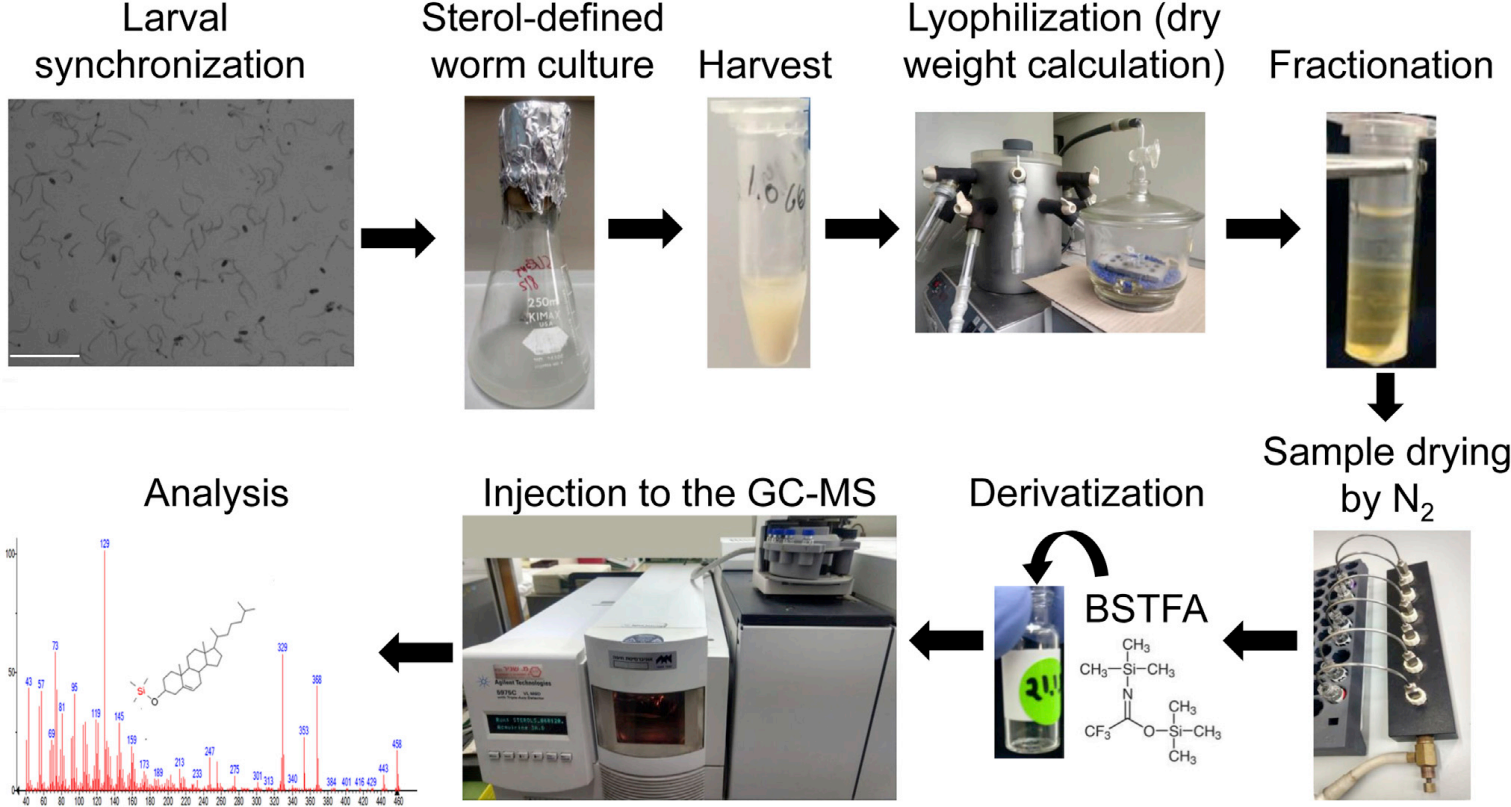
Schematic workflow of sterol studies of *C. elegans* Cultures of synchronized larvae grown in sterol-defined conditions are harvested, dried in a lyophilizer, and subjected to sterol extraction by hexane fractionation. Next, the organic phase of the extraction is dried using nitrogen and derivatized by N,O-Bis (trimethylsilyl) trifluoroacetamide (BSTFA). Finally, the samples are injected into a GC-MS instrument to identify and quantify sterols. Scale bar represents 500 micro micrometers.

The workflow described in this protocol (Figure 1) takes several days and requires the preparation of several solutions (see materials and equipment).

### Preparing of sterol-free *E. coli* bacteria as nematode food

**Timing: 3 days**

Sterol-free *E. coli* bacteria are prepared and used as the exclusive food of the nematodes throughout the experiment. Establishing a sterol-free *E. coli* culture is critical for achieving the end goal of culturing the nematodes with a single sterol of interest, without contamination from other environmental sterols.

1. Growing a culture of *E. coli* feed bacteria.
  ***Note:*** All the bacterial and *C. elegans* culturing steps should be performed under sterile conditions.
  a. Using a sterile inoculation needle, take a slur of *E. coli* NA22 bacteria from a −80°C glycerol stock and streak it on a 90 mm LB-agar plate. Grow for 16 h at 37°C. The plate can be stored for a maximum of one week at 4°C before use.
  b. In sterile conditions, using an inoculation needle, transfer 2 colonies from the plate to a 2-liter Erlenmeyer that contains a 500 mL MEM medium (see materials and equipment for preparation details). This will result in about 25 mL of concentrated nematode food. More Erlenmeyer flasks can be grown as needed.
    **CRITICAL:** Sterols are abundant in the environment. Use gloves, new glassware, and plasticware through the establishment and the harvest of the culture.
    ***Note:*** Although the bacteria are streaked on an agar plate traces of sterols from the plate are not affecting the culture of *C. elegans* because they are diluted in the large volume of the MEM medium and diluted again in the *C. elegans* culture.
  c. Grow the cultures for 20 h in a preheated 37°C shaker at 220 rounds per minute (henceforth, rpm).

2. Harvesting the bacterial culture.
  a. Pre-weigh centrifuge vessels that can hold more than 500 mL of liquid and mark the weight of each vessel.
  b. Transfer the bacterial cultures to the centrifuge vessels.
  c. Spin at 5,000 *g* for 30 minutes at 4°C.
  d. Carefully aspirate the soup without disturbing the bacterial pellet.
  e. Weigh the vessels again and calculate the weight of the pellet.
  f. Resuspend the pellets in S-basal medium (see materials and equipment for preparation details). One gram of wet bacterial pellet should be resuspended in 4 mL of S-basal medium.
    ***Note:*** It is easier to resuspend the pellet in 5 mL S-basal medium using a 5 mL pipette tip and, only once the pellet is completely dissolved, to add the rest of the S-basal medium to the centrifuge vessel.
  g. Transfer the suspension to a 50 mL sterile plastic tube and keep it at 4°C.
    **Pause point:** The concentrated bacteria can be used as nematode food for up to a month.

### Preparing synchronized, developmentally arrested *C. elegans* larvae

**Timing: four days**

This step aims to facilitate the establishment of a large population of developmentally arrested *C. elegans* larvae. This population is used to establish a synchronic culture of *C. elegans* that can be harvested at a specific point during the life cycle of *C. elegans* and analyzed by a GC-MS instrument in order to study stage-specific changes in sterol metabolism.

3. Growing *C. elegans* culture for bleaching.
  a. Establishing the culture: In a 250 mL Erlenmeyer set a culture of approximately 50,000 synchronized *C. elegans* larvae at the stage of L1 in 50 mL of S-complete medium (see materials and equipment for preparation details). This culture should be supplemented with 1 mL of concentrated nematode food and 50 μL of the stock solution (13 μM) of the sterol of interest. Grow for 3 days at 20°C while agitating the cultures at 175 rpm.
    ***Note:*** Hermaphrodite *C. elegans* is used through the protocol. In some nematode species, *C. elegans* included, liquid culture conditions may hinder male-female reproduction. Thus, it is advisable to use *C. elegans* hermaphrodites for experiments that require liquid cultures of worms (henceforth, “worm” means C. elegans).
  b. Harvesting: Once most of the animals reach the adult stage and have eggs in their uterus, transfer the culture to a 50 mL sterile plastic tube and place the tube in an ice bucket for approximately 30 minutes until most of the adults sink to the bottom (nematodes at other stages will mostly float).
  c. Washing: Carefully aspirate the upper phase without disturbing the pellet of adult nematodes. Add 40 mL of S-basal buffer to the pellet, place in an ice bucket for 30 minutes, and aspirate the upper phase. Repeat washing until the upper phase becomes clear (usually 4 washes).
  d. Transferring to microcentrifuge tubes: Use a diamond knife to break the end of a glass Pasteur Pipette and, using this pipette, transfer the pellet of adults to microcentrifuge tubes. Each tube should contain about 100 μL of the compact worm pellet.
    ***Note:*** In aquatic solutions, *C. elegans* nematodes adheres to plastic; thus, it is good practice to use a glass Pasteur Pipette rather than a plastic pipette for an optimal yield of the harvested cultures.

4. Bleaching to obtain a synchronized population of *C. elegans* arrested at the larval stage one (henceforth, L1 larvae).
  a. To each microcentrifuge tube from step 3d (containing adult *C. elegans*) add 1 mL of the bleach solution (see materials and equipment for preparation details), place the tubes in a vortex, and agitate for 8 minutes at maximal speed. Check for the complete disappearance of adult nematodes in the tubes by examining a drop of 10 μm from the tube under a dissecting scope. In case adult nematodes are still intact continue with the bleach until the completion of 10 minutes.
    **CRITICAL:** Household bleach is a hazardous chemical and should be used according to the manufacturer's safety guidelines. Household bleach should be aliquoted and stored at 4°C in the dark. The bleaching solution must be freshly prepared just before use.
  b. Centrifuge the tubes at 1,500 *g* for 2 minutes, carefully aspirate the soup without disturbing the pellet—a pyramid of white-colored *C. elegans* eggs.
  c. Add 1 mL of S-basal medium to the tube to break and wash the pellet, centrifuge the tubes at 1,500 *g* for 2 minutes, and aspire the liquid carefully. Repeat this wash two more times.
  d. After the third wash, add 1 mL of S-basal medium to the tube and transfer the tube content (S-basal medium with eggs post-bleach) to a clean 30 mL glass test tube.
  e. Leave the tubes on a shaker set to 175 rpm agitation for 24 h at 20°C. After this incubation period, all embryos should hatch and develop to the L1 larval stage and arrest due to lack of food and sterols, resulting in a synchronized population of animals.
    **CRITICAL:** Bleaching and incubation in S-basal medium should result in a culture of synchronized L1-arrested larvae without any carcasses or debris from the mothers. Such carcasses or debris can be a food source for the arrested L1s, resulting in their development and, consequently, the impaired synchronization of the culture. Moreover, efficient bleaching according to the guidelines above should result in a culture of live L1 larvae with no dead embryos and larvae. Before establishing the sterol-defined cultures, the level of embryonic and larval lethality should be monitored under a dissecting scope, and cultures with dead embryos and larvae should not be used.
    ***Note:*** L1 larvae in S-basal medium can survive up to 3 weeks, but it is advisable to continue with the protocol within 72 h post-bleach.

### Preparing of dietary-sterol stocks

The preparation of dietary-sterol stocks aims to minimize experimental noise by using the same sterol stock in all experiments.

***Note:*** For some sterols, it takes 24 h to completely dissolve in ethanol. Using stronger organic solvents like Dimethyl sulfoxide can harm *C. elegans* (Solis and Petrascheck, 2011) and therefore should be avoided. Thus, sterol stocks should be prepared at least 24 h before setting the cultures. All sterols used (see key resources table) should be dissolved in analytical-grade ethanol. The solubility of different sterols varies between 2.6 and 13 mM (see table about the solubility of sterols).

5. Preparing the stock sterols
  a. Dissolve the desired amount of sterol in ethanol.
  b. Leave at 4°C for 24 h until the powder completely disappears.
  c. Aliquots the sterols in microcentrifuge tubes and store them at −20°C for up to 3 months.

## Key resources table

**Table:** 

REAGENT or RESOURCE	SOURCE	IDENTIFIER
**Bacterial and virus strains**		

Feed bacteria *Escherichia coli:* strain NA22	Caenorhabditis Genetics Center	NA22 (RRID: WBStrain 00041948)

**Chemicals, peptides, and recombinant proteins**		

7-Dehydrocholesterol	Santa Cruz Biotechnology	Cat# sc-214398
Coprostanol	Sigma-Aldrich	Cat# C7578
Cholesterol	Sigma-Aldrich	Cat# 26732
Desmosterol	Cayman Chemical	Cat# 14943
Ergosterol	Sigma-Aldrich	Cat# 45480
Stigmasterol	Santa Cruz Biotechnology	Cat# sc-281156
β-Sitosterol	Sigma-Aldrich	Cat# S9889
Chloroform Amylene-free	Carlo Erba Reagents	Cat# 438613
Ethanol	BioLabs	Cat# 05250501
Methanol	BioLabs	Cat# 001368052100
Hexane	BioLabs	Cat# 08290601
N,O-Bis (trimethylsilyl) trifluoroacetamide (BSTFA) with 1% trimethylchlorosilane (TMCS)	Sigma-Aldrich	Cat# 155195

**Experimental models: organisms/strains**		

*Caenorhabditis elegans* (*C. elegans*): strain N2	Caenorhabditis Genetics Center	N2 (RRID: WBStrain00000001)

**Software and algorithms**		

Enhanced ChemStation E.02.02.1431 used to operate the GC-MS	N/A	https://www.agilent.com/en/products/software-informatics/massspec-workstations/gc-msd-chemstation-software
NIST MS Search 2.0 (Database NIST11) — used to identify compounds	N/A	https://chemdata.nist.gov/mass-spc/ms-search/

**Other**		

Gas chromatograph (GC)	Agilent	Model 7890A
Rxi-5Sil capillary column	Restek	Cat# 13623
Single-quad mass spectrometer (MS)	Agilent	Model 5975C

## Materials and equipment

**Table:** M9 salts (X5)

Ingredients	Final concentration (mM)	Amount
Na_2_HPO_4_ •7H2O	450.83	64 grams (g)
KH_2_PO_4_	110.22	15 g
NaCl	42.77	2.5 g
NH_4_Cl	93.47	5.0 g
Double distilled water (henceforth, ddH_2_O)	not applicable (henceforth, n/a)	Up to 1 liter
**Total**	**n/a**	**1 liter**

**Table:** Minimum Essential Medium (MEM)

Ingredients	Final concentration	Amount
M9 salts (x5)	n/a	200 mL
MgSO_4_ (1M)	2 mM	2 mL
CaCl_2_ (1M)	100 μM	100 μL
20% dextrose in ddH_2_O (1.1M)	22.22 mM	20 mL
ddH_2_O	n/a	to 1 liter
**Total**	**n/a**	**1 liter**

**Table:** S-basal medium

Ingredients	Final concentration (mM)	Amount
NaCl	100	5.85 g
K_2_HPO_4_	5.74	1 g
KH_2_PO_4_	44.09	6 g
ddH_2_O	n/a	to 1 liter
**Total**	**n/a**	**1 liter**

**Table:** Trace metals solution

Ingredients	Final concentration (mM)	Amount
Disodium EDTA	5	1.86 g
FeSO_4_ •7H_2_O	2.5	0.69 g
MnCl_2_ •4H_2_O	1	0.20 g
ZnSO_4_ •7H_2_O	1	0.29 g
CuSO_4_ •5H_2_O	0.1	0.025 g
ddH_2_0	n/a	to 1 liter
**Total**	**n/a**	**1 liter**

**Table:** S-complete medium

Ingredients	Final concentration (mM)	Amount
CaCl_2_ (1M)	3	3 mL
MgSO_4_ (1M)	3	3 mL
Trace metals	n/a	10 mL
Potassium citrate pH 6 (1M)	10	10 mL
S-basal	n/a	974 mL
**Total**	**n/a**	**1 liter**

**Table:** Bleach solution

Ingredients	Final concentration	Amount (mL)
Household bleach	60 mg/10 mL	2
KOH (5N)	0.5 N	1
ddH_2_0	n/a	7
**Total**	**n/a**	**10**

**Table:** Solubility of sterols

Sterol	Source	Cat#	Maximum solubility in ethanol (mM)	Final concentration in cultures (μM)
7-dehydrocholesterol	Santa Cruz Biotechnology	sc-214398	13	13
Coprostanol	Sigma-Aldrich	C7578	13	13
Cholesterol	Sigma-Aldrich	26732	13^a^	13
Desmosterol	Cayman Chemical	14943	13	13
Ergosterol	Sigma-Aldrich	45480	2.6	13
Stigmasterol	Santa Cruz Biotechnology	sc-281156	2.6	13
β-sitosterol	Sigma-Aldrich	S9889	2.6	13

^a^ Cholesterol forms white precipitates in ethanol. After 24 h at 4°C, the cholesterol is completely dissolved.

Preparation of solutions:

• M9 slates (X5): mix all ingredients, autoclave, store at ∼20°C indefinitely until required for use.
• MEM: mix all ingredients, filter through a 0.22 μm filter, store at 4°C indefinitely until required for use.
• S-basal medium: mix all ingredients, autoclave, store at ∼20°C indefinitely until required for use.
• Trace metals solution: mix all ingredients, filter through a 0.22 μm filter, store at ∼20°C in the dark indefinitely until required for use.
• S-complete medium: mix all ingredients, store at 4°C indefinitely until required for use.
• Bleach solution: prepare fresh every time by mixing all ingredients.

● Alternative: Many laboratories use NaOH in the bleach solution instead of KOH. In our experience, the use of KOH reduces the level of embryonic lethality during bleaching compared to NaOH, but we have not determined this quantitatively.
● Alternative: Some laboratories use bleach from Sigma-Aldrich (e.g., Cat# 425044), but we found that local household bleach works well; however, because of the variability of household bleach solutions, the effectiveness of each new batch should be tested.
● Alternative: In many protocols, the bleaching is carried out in 15 mL tubes, but we found that bleaching in microcentrifuge tubes is easier and has the same effectiveness.
● Alternative: Many laboratories use M9 medium instead of S-basal for the incubation of post-bleached embryos. We prefer incubation in S-basal because, in the subsequent experiment, the worms are cultured in an S-complete medium that is based on S-basal.

## Step-by-step method details

### Establishing sterol-defined *C. elegans* cultures

**Timing: 3 days—when done at 20°C**

This step aims to grow, for one generation (three days), a synchronized population of *C. elegans* in liquid culture supplemented with the sterol of interest or a vehicle (ethanol) control. The cultured *C. elegans* is harvested for downstream GC-MS analysis. A 100 mL of culture per condition should be prepared. Each such 100 mL culture gives approximately 330 mg of worm pellet that is sufficient for several GC-MS analyses.

1. Establishing the culture.
  a. To a 250 mL Erlenmeyer, add 100 mL S-complete medium, 2 mL from the NA22 bacterial food, the sterol of interest at 13 μM culture concentration, and 100,000 arrested, synchronized, and bacteria-free L1 larvae.
    **CRITICAL:** The synchronized L1-arrested larvae should not be used earlier than 16 h post-bleach or later than 72 h of incubation in S-basal at 20°C. Incubation shorter than 16 h may result in the miscalculation of the number of worms in the culture (see below), whereas culturing the L1 worms for more than 72 h may affect their survival, health, and the level of synchronization.
  b. Spit the 100 mL nematode culture into two 250 mL Erlenmeyer vessels (i.e., 50 mL into each vessel), cover the Erlenmeyer's opening with aluminum foil, and incubate on a shaker agitating at 175 rpm for about 3 days at 20°C.
    **CRITICAL:** The concentration of the worms in the culture should be about one worm per μl. More than three worms per μl may cause the worms in the culture to develop into the dauer diapause stage (Antebi, 2013), whereas cultures that are too diluted will result in an insufficient amount of worm pellet for the GC-MS analysis.

2. Harvesting, cleaning, and storing the cultured worms.
  a. After 48 h, check the developmental stage of the worms in order to harvest the culture when most worms are young adults with a few eggs. This point in time usually occurs around 72 h after the establishment of the culture but it can vary. Ideally, monitor the culture microscopically every 6 h.
  b. Transfer the culture to two new 50 mL sterile plastic tubes and place them in an ice bucket for 30 minutes until a pellet is visible—this pellet contains mostly adult worms.
  c. Carefully aspirate the liquid phase without disturbing the pellet. Add 40 mL of S- basal buffer, leave on ice for 30 minutes, and aspirate the liquid. Repeat until the upper phase becomes clear (usually 4 washes).
    ***Note:*** The washing steps aim to clean the adult worms from bacteria and enrich the culture for adult *C. elegans*, which sink more easily than eggs or young larvae.
  d. Add another 40mL of S-basal medium, seal the tube with a parafilm laboratory tape, place the tubes horizontally on a shaker and agitate at 175 rpm for 2 h at 20°C.
    ***Note:*** The two-hour incubation after the washing steps aims to clean as much as possible the alimentary canal of the worms from bacteria.
  e. Place the tubes in an ice bucket for 30 minutes, carefully aspirate the liquid without disturbing the pellet, add a further 40 mL of S-basal solution, and place the tubes in an ice bucket again as above.
  f. Repeat step 2e two more times.
  g. Transfer the worm pellet to pre-weighed 2 mL microcentrifuge tubes that have been pre-cooled in an ice bucket. Use a diamond knife to break the end of a Pasteur Pipette and, using this pipette, transfer the pellet of adults to the tubes. Each tube should contain about 500 μL of loose worm pellet.
    **CRITICAL:** The use of 2 mL microcentrifuge tubes is important for the extraction procedure (see below). Do not use the standard 1.7 mL tubes.
    ***Note:*** The 2 mL microcentrifuge tubes will be used for sterol extraction. In chemical analysis protocols it is commonly advised to use glassware rather than plasticware in order to avoid chemical contaminants. Nevertheless, to analyze a large number of small biological samples, the use of glass tubes might be challenging. To control for possible contaminants, it is highly advisable to use the same type of plasticware for blanks, standards, and samples. Moreover, the specific plasticware used should be tested for contaminants for each analyte and in case of contamination extraction should be conducted using glassware.
  h. Spin the tubes at maximal speed and immediately aspirate the liquid. Repeat centrifugation and aspiration until the tube contains only the compact worm pellet (about 300 μL in volume).
    **Pause point:** Samples can be stored at −80°C indefinitely until required for use.
  i. Uncap the tubes with the worm pellets and place them in a lyophilizer, weigh the pellets every hour until there is no more weight loss due to evaporation. In our Heto FD3 freeze dryer, this takes 2 h, but time may vary with vacuum strength.
  j. Weigh the tubes with the dry worm pellets and store them at −80°C until required for use. The dry weight of the pellet will be used to normalize the samples in the quantification step (Figure 6).
    **Pause point:** Lyophilized Samples can be stored indefinitely at −80°C until required for use.
    ***Note:*** To analyze worms that feed on a particular dietary sterol, worms should be cultured in the presence of this sterol for at least two generations in order for the mothers to transfer only the sterol of interest to the offspring. We usually grow cultures for ten generations on a specific sterol before the experiment. However, because *C. elegans* cannot grow to adulthood for more than one generation without sterols (Merris et al., 2004, Matyash et al., 2004), the culture of vehicle (ethanol) control worms can only be grown for one generation.
    ***Alternatives:*** Sucrose floatation is an alternative method to clean *C. elegans* cultures from bacteria, carcasses, and debris (Russell et al., 2020). However, sucrose floatation typically results in the loss of many adult worms. Moreover, the high osmolarity of sucrose may affect downstream procedures like sterol extraction. Thus, it is advisable to use gentler washing procedures as described here, rather than sucrose floatation.

### Lipid extraction and derivatization

**Timing: 6 h**

In this step, lipids are extracted from the dry pellets of *C. elegans* and the extracts undergo derivatization to enable the detection of sterols by the GC-MS instrument.

**Figure 3:**
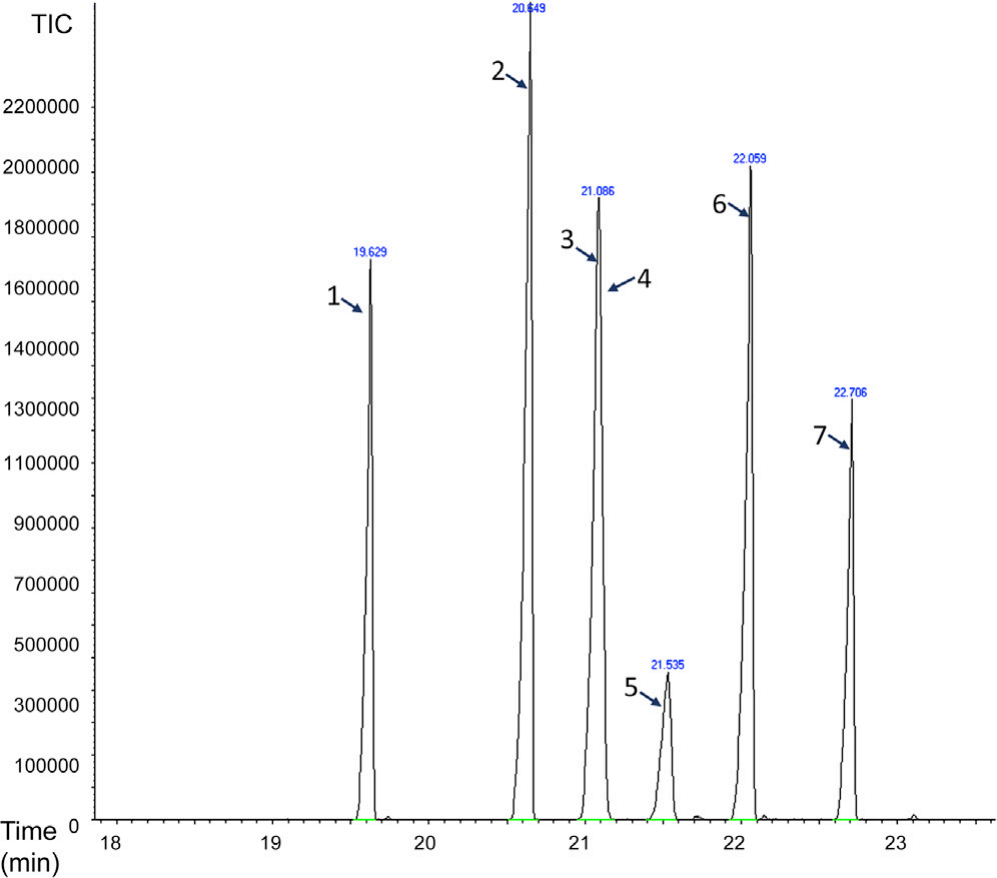
Representative GC-MS chromatographs of a mix of extracted standard sterols For GC-MS analysis we choose standards for the primary plant, fungal, and animal sterols as well as some intermediates of sterol metabolism in *C. elegans* and an internal standard (Coprostanol). 1. Coprostanol; 2. Cholesterol; 3. Desmosterol; 4. 7-dehydrocholesterol; 5. Ergosterol; 6. Stigmasterol; 7. β-sitosterol.
  b. GC details (these may vary by the type of the GC-MS instrument and column used): we used a gas chromatograph (Agilent Technologies, 7890A), equipped with a Rxi-5Sil MS capillary column (Restek, 13623). The GC was coupled to a single-quad mass spectrometer detector (Agilent technologies, 5975C).
  c. Program details: set the oven temperature to 70°C, raising to 250°C at a rate of 20°C per minute, and then from 250°C to 320°C at 10°C per minute, and then hold at 320°C for 10 minutes. Set injector temperature to 280°C and let the carrier gas (helium) flow at 1 mL/minute^−1^. Use a splitless injection with a purge time of 1 minute to inject a 2 μL volume of each sample by an auto-sampler.
    **CRITICAL:** The injector temperature should be higher than 250°C for the sterols to evaporate.
  d. Set the mass spectrometer transfer line to 320°C, the ion source temperature to 230°C, and the single-quad mass spectrometer detector temperature to 150°C in a scan mode, from 40 to 550 m/z.
    ***Note:*** If peak fronting or tailing appears, samples can be injected in split mode, although this could result in a lower sensitivity of detection and the loss of the detection of minor peaks, such as the 7-dehydrocholesterol in Figure 2.

3. Saponifying the samples
  a. Subject the pellets to three cycles of −80°C freezing and room-temperature thawing to break the cuticle of the worms.
  b. To each sample, add 500 μL of analytical-grade ethanol containing 4 μg of the internal standard; in our protocol, the internal standard is coprostanol (Merris et al., 2004).
    **CRITICAL:** It is critical to use internal standards to assess the efficacy of the extraction, derivatization, and detection steps. Not only is an internal standard essential for the comparison of the levels of sterols between samples, but it is also required for quantitative studies of sterols in the same sample (Mitsche et al., 2015). Before using a specific internal standard, perform one GC-MS analysis of the standard to find the retention time of the internal standard peak. Next, a worm sample should be analyzed to confirm that the internal standard is absent in worms. Finally, a *C. elegans* sample with the added internal standard should be analyzed to ensure that the peak of the internal standard does not overlap with any of the peaks of sterols of interest (for example, cholesterol in Figure 2).
    **Figure 2:**
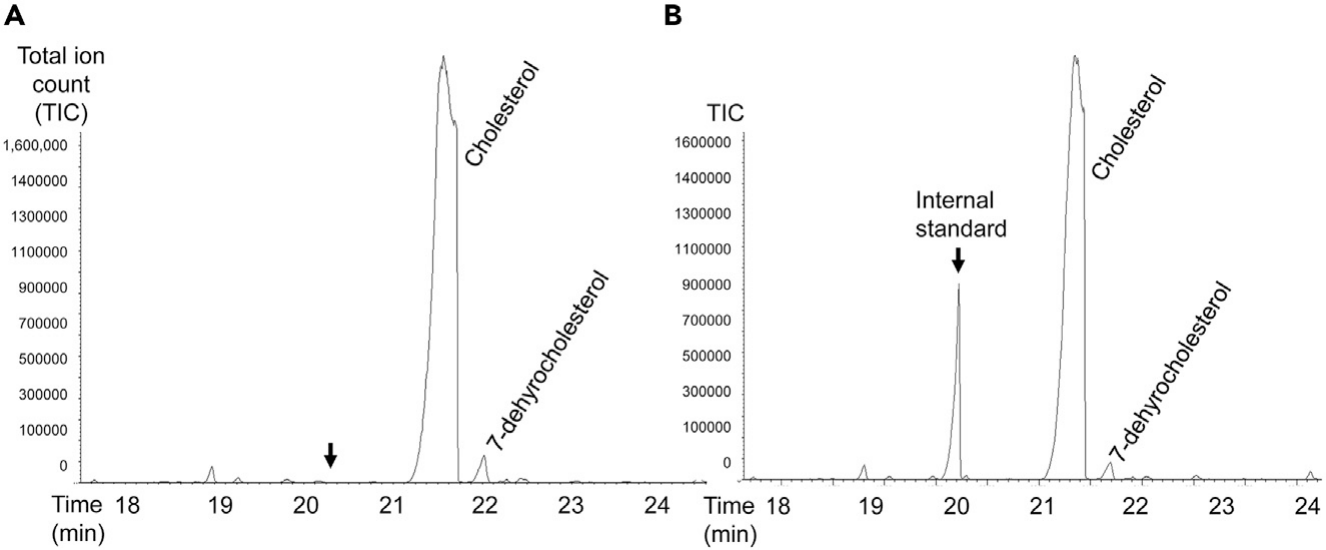
Representative GC-MS chromatographs of worm samples with and without an internal standard (A) *C. elegans* samples without any internal standard. (B) *C. elegans* samples with the coprostanol internal standard. No endogenous coprostanol is detected (arrow in A) and the retention time is different between the internal standard and *C. elegans* sterols as shown in panel B (see Figure 3 for more sterols).
  c. Add 50 μL of freshly made 10N aqueous NaOH to each 2 mL tube. Secure the cap of each tube with a microcentrifuge tube holder and incubate the tubes at 70°C for 1 h. Next, move the tubes to a 43°C water bath for three minutes.
    **CRITICAL:** Securing the tube caps is critical to protect the samples from a spillover caused by ethanol that evaporates at 70°C. Alternatively use screw-cap tubes.

4. Extracting lipids from the sample
  a. After the tubes are cooled to ∼20°C, open carefully to avoid spillover, and add 250 μL of ddH_2_O to each tube, mix and add 500 μL of hexane.
  b. Cap and seal the tubes with a parafilm laboratory film, vortex for one minute, and spin at 4,000 *g* for five minutes.
  c. Using a tip, carefully collect the upper (organic) phase of each tube, about 500 μL in volume, and transfer to a new 2 mL microcentrifuge tube. Repeat steps 4a–c two more times, combining the organic phases.
  d. Add 500 μL of water to each tube that contains the organic phase, and repeat fractionation as in step 2b. This step washes the hexane fraction from carryovers.
  e. Carefully aspirate the hexane upper phase and transfer to a new gas chromatography glass screw-thread vial (e.g., 9 mm wide vials).
    **CRITICAL:** Be sure not to take any of the lower aqueous phases because water is a strong inhibitor of the derivatization reaction.
  f. Dry the hexane with N_2_ gas flow in a fume hood. Typically, sterols are dried out as white sediments on the walls just above the bottom of the vial.
    **CRITICAL:** Be sure that no water in any form (drops, vapor, etc.) is left in the tube because the derivatization process is inhibited by water.

5. Derivatization of the lipids
  a. Add 50 μL of BSTFA to each sample and cap the vials with PTFE/silicon caps.
    **CRITICAL:** The BSTFA should be fresh for the optimal derivatization of substrates.
  b. Incubate the vials in an 80°C dry block for 30 minutes.
  c. Add 150 μL of hexane to each vial, mix to dissolve sterols that are on the vials' walls, and cap the vials. Preferably, samples should be analyzed immediately in the GC-MS instrument.
    **Pause point:** It is advisable not to pause during the extraction and derivatization steps. If necessary, samples after derivatization can be stored at −20°C for up to 3 days before the GC-MS analysis (longer storage may result in sample degradation or solvate evaporation).

### GC-MS analysis

**Timing: 2 h per sterol**

The GC-MS analysis aims to identify specific sterols in samples of interest and to quantify sterol levels within and between samples.

**Figure 4:**
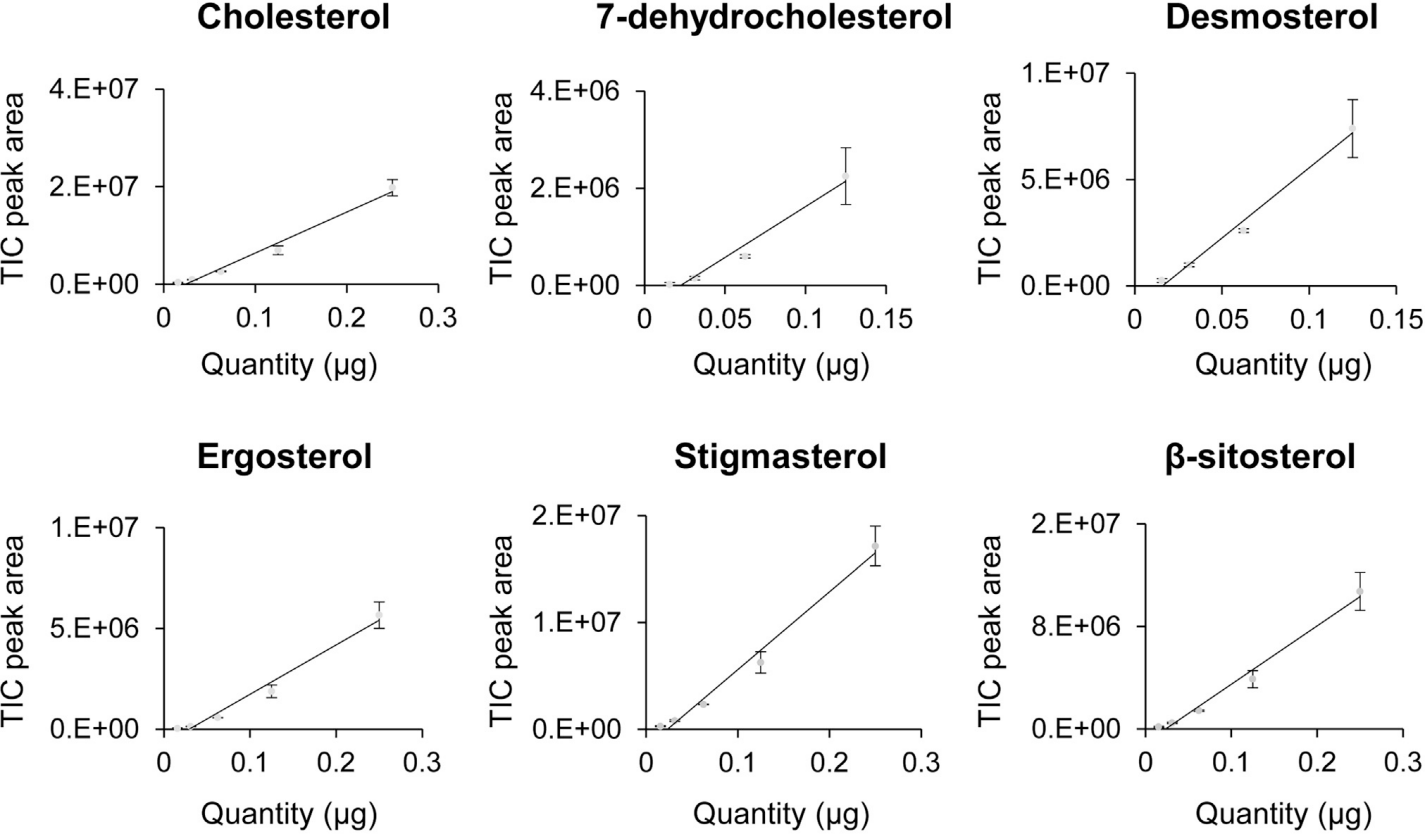
Calibration curves of different standard sterols Sterols were weighed and dissolved in ethanol and diluted to the designated quantities. Each calibration curve is based on three independent experiments. Error bars represent standard deviation (Std.) that for some points are too small to be visualized as bars in the graphs.

6. Building a GC-MS program for the separation of the different sterols
  a. Using a mix of sterol standards, build a GC-MS program for the separation and characterization of sterols (Figure 3).

7. Identifying and quantifying different sterols.
  a. Determine the retention time (the time where the molecule leaves the column and reaches the detector) of each sterol. Comparison to the retention time of known standards can reveal the identity of the sterols of interest (Figure 3).
  b. Identification by retention times, however, should be further validated through fragmentation patterns. MS analysis involves the breakages of the molecules that reach the detector to smaller ion fragments. Dependent on the original structure of the molecule, this fragmentation pattern can be used to distinguish between different molecules with the same retention time (for example 7-dehydrocholesterol and desmosterol (Figure 3 and step 7f)). Validate sterol identity by comparing the fragmentation patterns of the sterols of interest obtained by scan mode screening from 40 to 550 m/z (step 6d), with published reference spectra.
  c. Build a calibration curve for every sterol of interest by dissolving its purified commercial standard in ethanol in a designated quantity. In our GC-MS instrument, we use a range between 7.81 and 125 μg/mL but the exact range is instrument-dependent. Calculate the peak area for every concentration, build a calibration curve (Figure 4), and calculate the calibration equation for each sterol (Table 1).
    ***Note:*** As expected, the GC-MS analysis shows that the supplemented dietary sterol is the predominant peak in each sample but peaks of endogenous sterols are also detected (Figure 6).

**Table 1:** Calibration equations for the different sterols

	Sterol	Equation	R^2^
A	Cholesterol	Y=5E+07X-1E+06	0.987
B	Desmosterol	Y=7E+07X-1E+06	0.982
C	7-dehydrocholesterol	Y=2E+07X-471776	0.969
D	Ergosterol	Y=2E+07X-725537	0.978
E	Stigmasterol	Y=7E+07X-2E+06	0.985
F	β-sitosterol	Y=5E+07X-1E+06	0.985

The calibration equations were obtained from the calibration curves (Figure 4) of three independent GC-MS analyses.
  d. For every extraction procedure, calculate by GC-MS analysis the level of the internal standard before and after extraction to estimate the extraction efficiency.
  e. Quantify the level of each sterol of interest by placing the value of its peak area in the line equation obtained from the calibration curve (Figure 4 and Table 1). Correct the value according to the extraction efficiency of the internal standard and divide the value by the dry weight of the sample (see Table 2 and Figure 6).
    **CRITICAL:** If the peak area of a given sterol is outside the range of the calibration curve sterol concentration cannot be calculated accurately. In this case, dilute or concentrate the sample to fit the range of the calibration curve.
    ***Note:*** In some studies, normalization is based on the endogenous level of a single or group of biomolecules (e.g., total phospholipids). However, because changes in sterols metabolism can affect the level of many different metabolites including phospholipids it is better to normalize to dry weight.

**Table 2:** An example of the calculation of the quantities of extracted sterol standards according to the peak areas obtained by the chromatographs in Figure 3

	TIC peak area	Extraction efficiency (%)	Quantity (μg)	Corrected quantity (μg)
Coprostanol (Internal Standard)	4549529	0.79		
Cholesterol	7323398		0.12	0.15
Desmosterol	5582508^a^		0.94	1.19
7-dehydrocholesterol	2427466^a^		0.14	0.18
Ergosterol	1918794		0.13	0.17
Stigmasterol	6304675		0.12	0.15
β-sitosterol	3686069		0.09	0.12

**Figure 5:**
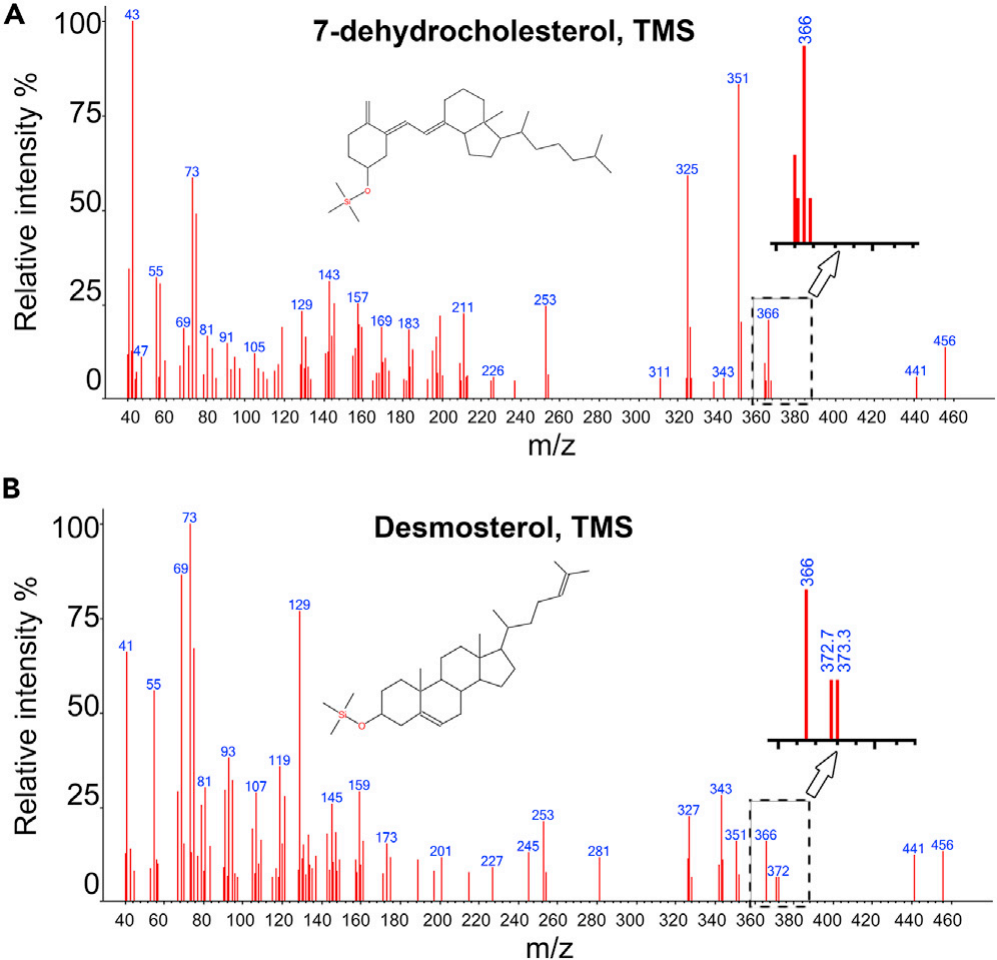
Fragmentation pattern and the identification of specific MS ions for desmosterol (A) The fragmentation pattern of 7-dehydrocholesterol. (B) Analysis of the fragmentation pattern of desmosterol reveals two ions, 372.7 and 373.3 that are not detected in 7-dehydrocholesterol (inserts in A and B). We used these specific ions to calculate the levels of the two sterols (step 7f). Specific ions were selected only if they were separated from main ions mass in more than 0.05 units. For example, the specific ions around 366 in 7-dehydrocholesterol (insert in A) cannot be used to distinguish it from desmosterol because they are too close to the mass of the 366 ion.

The calculation of quantities is based on the peak area of each sterol and its calibration curve equation (Table 1), divided by the extraction efficiency of the internal standard.

a Areas of desmosterol and 7-dehydrocholesterol were estimated as described below.
  f. The peaks of desmosterol and 7-dehydrocholesterol overlap (Figure 3) preventing their distinction by retention time. However, the analysis of pure standards of the two sterols in scan mode revealed two ions of M/Z ratio 372.7 and 373.3, detected in desmosterol but not in 7-dehydrocholesterol (Figure 5). A constant ratio of 0.0042 between the accumulative area of the desmosterol-specific peaks and total ions peak (Table 3) was determined. This ratio allows the estimation of the desmosterol fraction of the shared peak. For example, in Figure 3, the total-ion peak area is 8,009,974. The area of the same peak when only the 372.7 and 373.3 ions are accounted for is 23,612. Using the 0.0042 ratio, the desmosterol-specific peak area is calculated to be 5,582,508 (23,612/0.0042). The subtraction of the peak area of desmosterol from the area of the total peak reveals the peak area of 7-dehydrocholesterol (8,009,974 - 5,582,508 = 2,427,466), resulting in 1.19 μg and 0.18 μg respectively (Table 2). We used the National Institute of Standards and Technology NIST11 library (Table 4) as a source for fragmentation patterns of standard sterols.

**Table 3:** Calculation of desmosterol and 7-dehydrocholetserol levels

Biological replica	Desmosterol- specific ion 372.7 area	Desmosterol- specific ion 373.3 area	Ions 372.7+ 373.3 area	Desmosteroltotal-ions area		Specific-ions area/total-ions area
1	23,999	11,329	35,328	7,396,564		0.0048
2	12,401	6,578	18,979	4,576,532		0.0041
3	35,262	12,745	48,007	1,3150,659		0.0037
4	43,308	20,818	64,126	14,759,511		0.0043
					Avg.	0.0042
					Std.	0.00046

The ratios of peak areas were obtained by subtracting the desmosterol-peak area from the total-peak area. This ratio enables the calculation of the amount of both desmosterol and 7-dehydrocholesterol in the samples.

**Table 4:** Characterization of the sterols identified and quantified using the GC-MS analysis

	Trivial name	Systematic name	MF; MW (g/mol)	Sterol structure	Sterol ester structure (after derivatization)	Similarity to database NIST11 (%)	Retention time (min)	Major ions (*m/z*) resulted from derivatized sterols by order of prevalence (high to low)
1	Coprostanol	5β-cholestan-3β-ol	C_27_H_48_O; 388.7			44.5	19.60	370, 55,75,215,43,371,57,55,73,95

2	Cholesterol	Cholest-5-en-3β-ol	C_27_H_46_O;386.7			80.2	20.60	129,329,73,43,368,75,57,95,458,41

3	Desmosterol	Cholesta-5,24-dien-3β-ol	C_27_H_44_O;384.6			92.3	21.08	69,129,73,75,41,55,343,81,95,119

4	7-dehydro-cholesterol	Cholesta-5,7-dien-3β-ol	C_27_H_44_O;384.6			88.1	21.08	351,325,73,352,43,366,456,143,326,75

5	Ergosterol	24-methylcholesta-5,7,22E-trien-3β-ol	C_28_H_44_O;396.6			95.7	21.50	363,43,73,69,55,337,75,41,157,81

6	Stigmasterol	Stigma-5,22-dien-3β-ol	C_29_H_48_O;412.7			90.3	22.05	83,129,55,73,69,75,81,43,484,133

7	β-sitosterol	5-stigmasten-3β-ol	C_29_H_50_O;414.7			96.2	22.70	129,357,396,43,73,57,381,486,75,95


Table columns are sterol names; molecular formula (MF) and molecular weight (MW; g/mol); molecular structures before and after derivatization; percentages of similarity to the NIST11 database; retention times; and major ions detected in the GC-MS analysis (m/z).

## Expected outcomes

**Figure 6:**
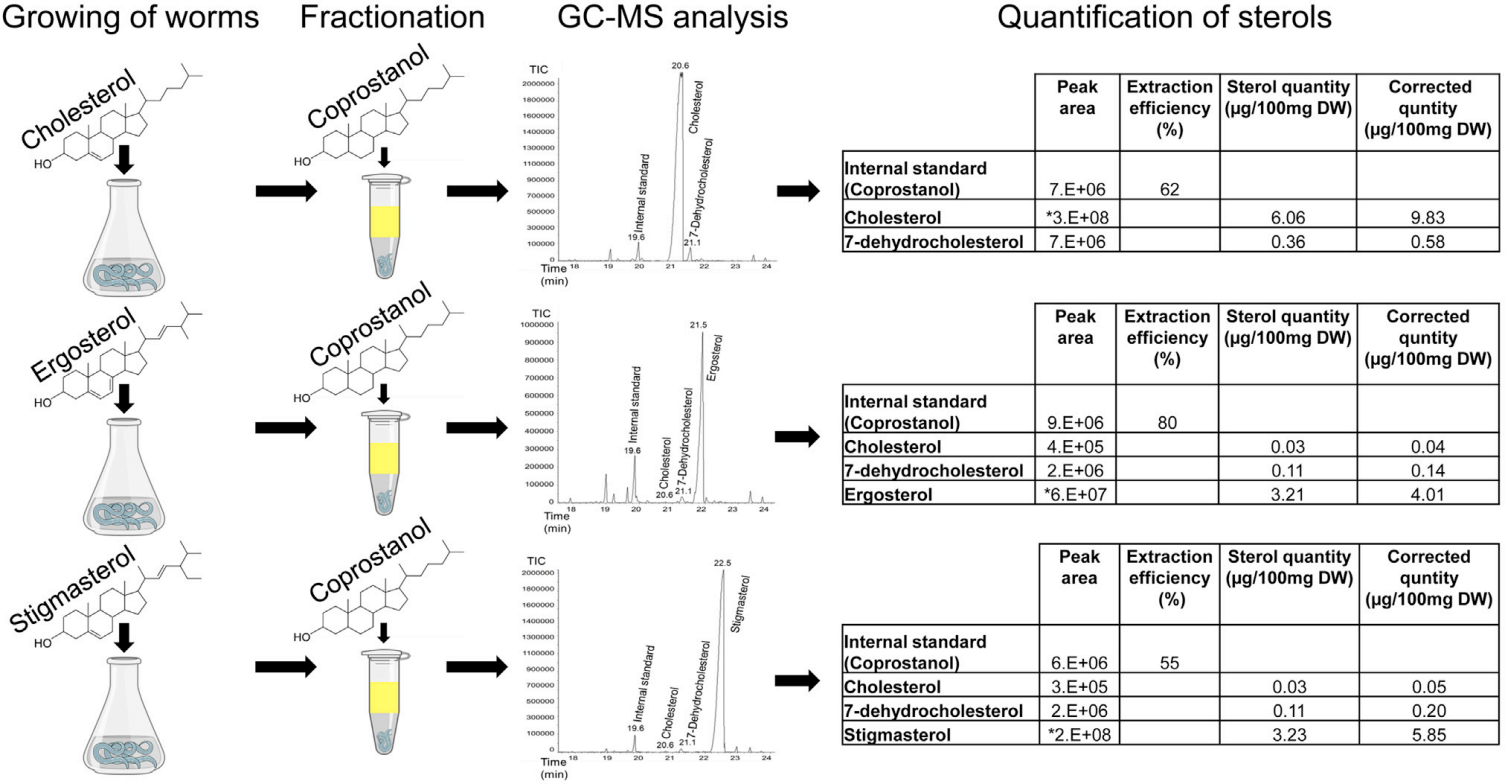
Quantification of sterols from *C. elegans* cultures Synchronized cultures that were grown with cholesterol, ergosterol, or stigmasterol were analyzed. Before the fractionation step, an internal standard was added to each sample. The different sterols, based on their peaks, were identified by the retention time and the patterns of their fragmentation were compared to the NIST11 database in order to validate sterol identity. The absolute quantity of each sterol was calculated by placing peak areas into the calibration curves equation (Table 1) and the resulting value was then multiplied by the extraction efficacy. The “∗” sign indicates the concentration of analytes that did not fit the calibration curve range. In these cases, samples of analytes were diluted or concentrated until they fit into the calibration curve and the results were multiplied by the dilution/concentration factor. “DW” is the dry weight of worm pellets. Desmosterol and b-sitosterol that appear as standards in Figure 3 are not detected because b-sitosterol is not part of the dietary sterols of the cultures and the levels of desmosterol in *C. elegans* are below the detection level of our instrument (Shamsuzzama et al., 2020).

The expected outcome of the protocol is the testing of the ability of *C. elegans* to utilize different sterols and the detection, identification, and quantification of *C. elegans* sterols. By growing *C. elegans* in sterol-defined cultures, the supplementation of the cultures with sterols of interest, and GC-MS analysis the conversion of dietary sterols to cholesterol can be determined *in vivo* (e.g., Figure 6). Moreover, this method opens an avenue into the study of sterol metabolism in the different physiological and developmental states of *C. elegans.* For example, this method facilitates the determination of the composition and level of different sterols in the dauer larval stage of *C. elegans* and other larval stages. Moreover, our protocol lays the foundation for the development of protocols for genetic and biochemical studies of sterol metabolism in other nematodes by GC-MS analysis.

## Limitations

Endpoint analysis without kinetics: GC-MS analysis is designed to determine the composition and levels of sterols at a specific endpoint of an experiment. In theory, it is possible to collect samples at various points in time to study the kinetics of the metabolism of sterols (Dabrowski et al., 2020). However, this approach does not support the direct analysis of metabolic flux, which can be studied by labeling sterols and their precursors by heavy atoms (Mitsche et al., 2015; Mitsche et al., 2015) which is beyond the scope of this protocol.

Efficiency of extraction: The efficiency of extraction can vary between samples and even with regards to different sterols in the same sample (Ms et al., 2018). Thus, the use of at least one internal standard and a mix of standards of the sterols of interest is critical for relative quantitative analysis of changes in sterol levels.

Derivatization: For GC-MS analysis, sterols must be derivatized to increase their volatility (Prabhu et al., 2017). Differences in the levels of derivatization of various sterols in the same sample or between samples can affect data quantification, interpretation, and reproducibility. The use of Liquid Chromatography-Mass Spectrometry (LC-MS) enables the analysis of sterols from biological samples without derivatization (Skubic et al., 2020, Qiu et al., 2021) however, the analysis of sterols by LC-MS has other limitations (Jenner and Brown, 2017).

## Troubleshooting

### Problem 1

Contamination of the sterol-defined cultures (step 1 in the step-by-step method details) by environmental sterols. To verify that no background sterols contaminating the experiment a sterol-free culture of *C. elegans* must be established in every experiment. Sterol-deprived phenotypes such as the developmental arrest of the F2 progeny larvae in the control culture indicate the lack of sterols in the culture whereas the development of these larvae into adults indicates sterol contamination.

### Potential solution

All the components of the experimental system must be replaced. Special care should be taken not to use any vessel, medium, or material that may contain traces of sterols. A preliminary experiment to validate that the culture is sterol-free is a requisite step before conducting any experiment.

### Problem 2

Unsynchronized cultures (step 2 in the step-by-step method details). The synchronization of cultures is important for the analysis of stage-specific sterol metabolism. One primary reason for unsynchronized cultures is inefficient bleaching, leaving carcasses or debris that can be a food source for the L1s.

### Potential solution

Optimize the bleaching reaction to completely dissolve the bodies of the parental *C. elegans* nematodes.

### Problem 3

Within each sample, variation in the levels of fractionation, derivatization, and detection may affect the absolute and relative quantification of the different sterols (steps 3–7 in the step-by-step method details).

### Potential solution

To circumvent the above caveat, a mixture of known amounts of commercial pure standards (above 95% of purity) should be fractionated, derivatized, and analyzed the same way as the biological samples. The derivatization of standards is used to unveil the efficacy of fractionation and derivatization, as well as the level of detection of each sterol by GC-MS.

### Problem 4

Between samples, differences in the levels of fractionation, derivatization, and detection of the same sterol (steps 3–7 in the step-by-step method details).

### Potential solution

An internal standard should be added to each sample. Quantitative analysis of the area of peaks of the different sterols should be normalized to the peak area of the internal standard to normalize variations between experiments, similar to the analysis shown in Figure 6.

### Problem 5

Lack of detection of sterol peaks in the GC-MS analysis (step 7 in the step-by-step method details). Because the GC-MS analysis is the endpoint of the experiment, a lack of signal can stem from the metabolization of the specific sterol, as well as from problems in extraction, derivatization, or GC-MS operation/detection. Thus, pinpointing the problem to a specific intermediate step is critical.

### Potential solution

Different controls can pinpoint the step in which the problem arose:

The use of internal sterol standards. Adding internal sterol standards, like coprostanol, to the samples not only normalizes the detection but also can reveal whether the extraction, derivatization or GC-MS operation/detection took place in a way that enables the detection of sterols in the samples.
The use of a mix of sterol standards as a positive control for the extraction, derivatization, and detection steps.

If the internal standard and/or the mix of sterol standards are not properly detected/quantified, each of the steps of the procedure should be tested separately:

Test GC-MS settings and detection by running known sterols standards that did not undergo fractionation.
Test derivatization by derivatizing commercial sterol standards and running a GC-MS analysis along with standards that do not require derivatization for detection (i.e., non-sterol standards that have a lower boiling point and higher thermal stability).
Test for fractionation efficiency. In case the above tests for GC-MS detection and derivatization efficacy did not identify the problem, the efficiency of fractionation can be tested by determining the levels of the sterol standards before and after fractionation.

## Resource availability

### Lead contact

Further information and requests for resources and reagents should be directed to and will be fulfilled by the lead contact, Amir Sapir (amir-s@sci.univ.ac.il).

### Materials availability

This study did not generate new unique reagents.

### Data and code availability

This study did not generate any unique datasets or code*.*
